# A Study of the Coupling of FET Temperament Traits with Major Depression

**DOI:** 10.3389/fpsyg.2016.01848

**Published:** 2016-11-25

**Authors:** Irina N. Trofimova, William Sulis

**Affiliations:** Collective Intelligence Laboratory, Department of Psychiatry and Behavioral Neurosciences, McMaster University, HamiltonON, Canada

**Keywords:** Major Depression, temperament profiles, DSM/ICD descriptors, FET framework, sex differences, age differences

## Abstract

**Objective:** Temperament and mental illness have been linked to the same systems of behavioral regulation. A temperament model, carefully structured to respond to subtle differences within systems of behavior regulation, should exhibit distinct temperament patterns in the presence of mental illness. Previous comparisons of temperament profiles in mental disorders used mostly emotionality-related traits. In contrast, the Functional Ensemble of Temperament (FET) model differentiates not only between emotionality traits, but also between traits related to physical, verbal, and mental aspects of behavior and maps 12 functional aspects of behavior to temperament traits as well as to symptoms of mental illnesses. This article reports on the coupling of sex, age, and temperament traits with Major Depression (MD) using the FET framework.

**Method:** Intake records of 467 subjects, ages 17–24, 25–45, 46–65, 66–84 were examined, with temperament assessed by the Structure of Temperament Questionnaire (based on the FET).

**Results:** The presence of MD was associated with changes in mean temperament scores on 9 of the 12 traits. The results were in line with the DSM-5 criteria of fatigue (patients with MD reported a significant decrease in three types of endurance – motor-physical, social-verbal, and mental), of psychomotor retardation (a significant decrease in physical and social-verbal tempo) and of worthlessness (as low Self-Confidence). The results also showed that three new symptoms, high Impulsivity, high Neuroticism, and diminished Plasticity, should be considered as depressive symptoms in future versions of the DSM. As a significant negative result, no interaction of age or sex (with the exception of the Self-Confidence scale) with MD was found for temperament traits.

**Conclusion:** The value of differentiating between physical, social, and mental aspects of behavior is demonstrated in the differential effects of major depression and gender. The value of differentiating between endurance, dynamical and orientation-related aspects of behavior is also demonstrated. The deleterious impact of MD on temperament scores appeared to be similar across all age groups. The appearance of high impulsivity, neuroticism, and low plasticity deserve further study as associated factors in future versions of the DSM/ICD.

## Introduction

### Temperament and Mental Illness Lie along a Continuum

Clinical psychology and psychiatry are rather young sciences. As a result, research into the underlying neurophysiological systems, as well as systematic principles for the classification of mental disorders, is still in its early stages. Over the past several decades, investigations into the functional neuroanatomy and neurochemistry associated with various mental disorders have resulted in a large number of diverse, complex, and conflicting reports. Studies in neuropharmacology and neurochemistry have shown that nearly all of the disorders listed in the DSM and ICD classifications respond to medications manipulating various neurotransmitter systems. Responses to such psychopharmacological treatment are variable and in many cases only modest, and much more work needs to be done in this line of research. Nevertheless, development of the next generation of classification systems for mental disorders would benefit from a consideration of temperament models grounded upon research into the neurochemistry of behavioral regulation. This paper offers a framework for the description of psychological systems of behavioral regulation.

A temperament model, carefully structured to reflect subtle differences among neurotransmitter systems of behavioral regulation (emerging as temperament), should exhibit distinct profiles in the presence of illness consistent with DSM-5 symptoms of such illness. Temperament is understood as arising from individual differences in neurochemically based systems of behavioral regulation (Rothbart, 1988; Bates and Wachs, 1994; Kagan, 1994; Strelau, 1998; Rusalov and Trofimova, 2007; Zentner and Shiner, 2012). Since the time of Hippocrates and Galen, associations have been suggested between temperament and mental illness, continuing into the present in the idea of the affective temperaments (Rihmer et al., 2010; Rovai et al., 2013). The focus on temperament in the context of the analysis of the regulatory systems underlying mental illness is based the premise of a common etiology underlying temperament and mental disorders (i.e., neurochemical systems of behavioral regulation). In fact, multiple temperament traits (such as impulsivity, sensation seeking, neuroticism, endurance, plasticity, sociability, or extraversion) have been linked to brain neurotransmitters and hormonal systems, i.e., the very same systems implicated in mental disorders (Gray, 1982; Cloninger, 1986; Heath et al., 1994; Kagan, 1994; Depue and Morrone-Strupinsky, 2005; Rusalov and Trofimova, 2007; Zentner and Shiner, 2012; Trofimova, 2016a; Trofimova and Robbins, 2016). Thus temperament and mental illnesses appear to represent varying degrees along the same continuum of behavioral regulation (Clark et al., 1994; Heath et al., 1994; Mehrabian, 1995; Ball et al., 1999; Weinstock and Whisman, 2006; Brown, 2007; Weiss et al., 2009; Karam et al., 2010; Trofimova and Sulis, 2010).

Much of the research in this area focused on temperament models and traits related primarily to emotionality, such as Negative Affect (Watson et al., 1988; Clark et al., 1994; Mineka et al., 1998), Harm Avoidance (Pelissolo and Corruble, 2002; Nery et al., 2009; Kampman and Poutanen, 2011; Kampman et al., 2012; Lu et al., 2012), Neuroticism (Costa et al., 2005; Kotov et al., 2010; Klein et al., 2011), and depressive affective temperament (Akiskal, 1989; Rihmer et al., 2010). These results showed an ability of affect-based temperament models to reflect the symptoms of low mood and feelings of worthlessness in Major Depression (MD). A recently proposed reconceptualization of the diagnostic categories for mood and anxiety disorders for the DSM-5 (Insel, 2014) is also based on temperament models with dimensions of Negative and Positive Affects (Clark and Watson, 2006).

Affect-oriented temperament models, however, appear to be very insensitive in differentiating between various types of mental disorders.

Far fewer studies have investigated the coupling between depression and non-emotionality traits, in spite of the fact that the DSM-5 considers a broad range of symptoms: fatigue, poor attention, memory, and irritability, dysfunction of basic regulatory systems such as sleep, appetite, energy, and alterations in the dynamical aspects of psychological functions manifesting in psychomotor retardation (sometimes agitation), poor concentration, lethargy (or restlessness), and a motivation. Insufficient attention to functional aspects and a primary focus on emotionality in earlier temperament models may explain why the findings from previous studies examining temperament profiles in depression could not be easily mapped onto DSM/ICD descriptors.

Studies of non-emotionality traits using the Five Factor model reported a decrease in extraversion in depression and an increase of extraversion in mania (Costa et al., 2005; Kotov et al., 2010; Klein et al., 2011). Studies using Gray’s Reinforcement Sensitivity model showed links of depression to lower Behavioral Activation and higher Behavioral Inhibition (Naragon-Gainey et al., 2013). Studies using Cloninger’s Temperament Character Inventory (TCI; in addition to links between depression and high scores on the TCI Harm Avoidance scale) found reduced scores on the TCI Self-Directedness scale (Hansenne et al., 1999; Pelissolo and Corruble, 2002; Nery et al., 2009; Mochcovitch et al., 2012). Moreover, Nery et al. (2009) also included groups with depression in remission, and, in addition to the higher scores on TCI Harm Avoidance and lower Self-Directedness scales, they found higher scores on the Novelty Seeking, and Self-Transcendence scales and lower scores on the Reward Dependence and Cooperativeness scales of the TCI in depressed subjects. Remitted subjects, however, differed only in reporting lower Self-Directedness, in comparison to healthy participants. This suggests that there may be a state dependent effect of depression on the reporting of temperament traits. Using a variant of the Cloninger model, Elovainio et al. (2004) found elevations on the scales of Impulsiveness, Shyness to strangers, Fatigability, Sentimentality, and Persistence.

### The Functional Ensemble of Temperament (FET) Model

The FET framework organizes temperament traits and symptoms of mental illness in a 3 × 4 matrix categorized by the functional aspects of human behavior (Trofimova, 2016, Trofimova, submitted; Trofimova and Robbins, 2016). The precursor of the FET model, the Structure of Temperament Questionnaire (STQ), was developed by Rusalov within an experimental tradition for studying properties of nervous systems. This tradition started within differential psychophysiology more than a 100 years ago in Pavlov’s Research Institute and continued in Eastern Europe (Strelau, 1998; Rusalov and Trofimova, 2007 for review). Using human subjects, this school experimentally measured multiple behavioral and psycho-physiological indices, including EEGs and evoked potentials. This resulted in the first activity-specific model of temperament separating physical-motor and social-verbal traits of temperament (Rusalov, 1979, 1989; Rusalov and Trofimova, 2007). Rusalov’s model was recently revised (Rusalov and Trofimova, 2007; Trofimova, 2010a; Trofimova and Sulis, 2010, 2011) and was complemented using links to multiple neurochemical systems in the form of the FET model (Trofimova, 2016a; Trofimova and Robbins, 2016).

The FET model uses double-word names of the scales (Motor-physical or Social-verbal) but for simplicity throughout of the paper we will refer to these scales using single-word names, i.e., either Motor or Physical or Social, but the reader should keep in mind the underlying connotation.

**FIGURE 1:**
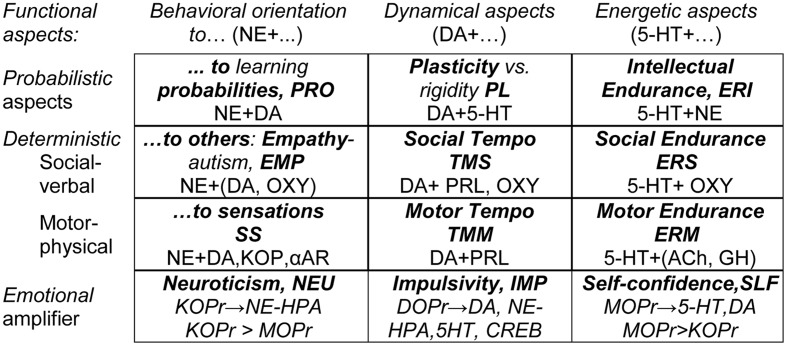
**The neurochemical model Functional Ensemble of Temperament (FET): 12 traits regulate specific functional aspects of behavior and are based on interacting neurotransmitter systems.** Bold font highlights names of the temperament traits and their abbreviations. 5-HT, serotonin; DA, dopamine; NE, norepinephrine; Ach, acetylcholine; GH, Growth Hormone; SOM, Somatostatin; PRL, prolactin; OXY, oxytocin; αAR – α-adrenoceptors, KOPr, MOPr, DOPr, κ-, μ-, and Δ-opioid receptor systems.

The neurochemical FET hypothesis linking the components of the STQ to ensembles of neurotransmitters is based on an extensive integration of research within neurochemistry and neuropharmacology. Space does not permit a detailed discussion of the neurochemical hypothesis underlying the FET, which has been described elsewhere (Trofimova, 2016a; Trofimova and Robbins, 2016). A summary of the hypothesis is presented in **Figure 1** that shows putative links between FET traits and various neurotransmitter and opioid receptor systems.

A distinct non-emotional symptom of depression is psychomotor retardation. Psychomotor retardation has been shown to be regulated mostly by dopaminergic systems (Bennabi et al., 2013). These, in turn, receive modulatory influences from μ-opioid receptor (MOPr, Johnston and North, 1992; Degli-Uberti et al., 1995) and Δ-opioid receptor systems (DOPr, Comer et al., 1993; Jutkiewicz et al., 2005). According to the FET model, dysfunction in the DOPr system should lead to dysregulation of dopaminergic systems, resulting in a slowing of the tempo of activity. This will manifest itself in a reported reduction in physical and social types of tempo (in the case of well learned behaviors) and in the plasticity of behavior (in the case of less predictable situations). Moreover, DOPr interaction with DA and MOPr systems appears to play an important role in the premature generation of an action (i.e., impulsivity; Comer et al., 1993; Broom et al., 2002; Jutkiewicz et al., 2005; Robbins, 2007; Olmstead et al., 2009; Pradhan et al., 2011). The FET model suggests that in depression, due to the impact of a dysregulation of MOPr and DOPr systems on DA release, behavior can become sluggish and less plastic. It will also bcome over-reactive toward occasional stimuli that would not normally trigger a behavioral reaction, leading to an elevation of impulsivity. Impulsivity is rarely discussed as a symptom of depression, however, a few studies reported high impulsivity in depressed patients (Elovainio et al., 2004; Trofimova and Sulis, 2010; Trofimova and Christiansen, 2016).

In regards to the emotionality-related traits of temperament and symptoms of depression, the FET suggests that a dysregulation in opioid receptor systems is expressed in several symptoms of depression. Opioid receptor systems regulate the release of monoamines, and their up- or down-regulation can trigger a dysregulation of serotonin and dopamine systems linked to depression. Thus, a dysregulation in the MOPr can produce the symptom of dysphoria (Degli-Uberti et al., 1995; Bodnar, 2011). Dysregulation in the κ-opioid receptor system can produce symptoms of low mobilization and low motivation and/or comorbid anxiety (Takahashi et al., 1990; Simonin et al., 1998; Filliol et al., 2000; Schwarzer, 2009). Dysregulation in the delta-opioid system, as described in the previous paragraph, can produce the symptoms of impulsivity and diminished plasticity of behavior.

It is most important to note that the FET model suggests that there is *no* one-to-one correspondence between neurotransmitter systems and individual temperament traits (or mental disorders). Instead, each temperament trait emerges from interactions within specific ensembles of neurotransmitter (and opioid) systems. The neurotransmitter (and opioid) systems contributing to each trait-related ensemble are given in **Figure 1**.

The STQ/FET model considers 12 temperament traits. Nine traits are associated with three formal functional aspects of behavior (energetic, dynamic, and orientational). Each functional aspect is further assessed in three domains (intellectual, physical, and social). In addition there are three traits related to emotionality (Neuroticism, Impulsivity, and Self-Confidence **Figure 1**).

It is also important to emphasize the distinction between the validation history of the STQ as a psychometric test, and the validation of the neurochemical model FET. Neurochemical models cannot be validated using the factor structure of psychometric tests and therefore the FET was validated via a review of the consensus in neurochemistry in regards to the role of neurotransmitters (Trofimova, 2016a; Trofimova and Robbins, 2016). Nevertheless, the psychometric properties of the scales of the STQ (which structure the subsequent FET model), have been extensively studied and validated over several decades (Rusalov and Trofimova, 2007; see also Trofimova, 2010a for a summary). The FET hypothesis linking neurotransmitter systems to temperament traits is supported by the extensive literature on the neurobiology of behavioral regulation, and several entire disciplines in science (neurochemistry, psychopharmacology) are examining such regulation (Trofimova, 2016a; Trofimova and Robbins, 2016 for reviews). No research group could be expected to verify the complete FET model in a single study due to the multiplicity of components and their complex relationships. Thus confirmation of the neurochemical components of the FET model was *not* a goal of this study.

A major goal of the FET program is the development of a systematic model of behavioral regulation, upon which to base a novel classification of a wide range of mental disorders. Temperament traits, as defined within the FET model, have been shown to react to the presence of generalized anxiety disorder (Trofimova and Sulis, 2016a) and other types of mental illness (Trofimova and Christiansen, 2016). The primary *objective* of the present study is to further the FET program by examining the coupling between MD and the temperament traits of the FET model. If the temperament profiles of depressed patients show a pattern consistent with the DSM descriptors, then this would provide a significant step forward in supporting the FET model as a framework with sufficient differential power upon which to base a new classification system.

By no means do we suggest that a temperament test should be a diagnostic tool for the assessment of mental disorders. We do, however, suggest that the structure of temperament and the structure of a taxonomy of mental illness should use the same framework, and that such a framework should reflect key aspects of human behavior and the most fundamental systems of behavioral regulation.

This study reports on an investigation of features of depression across multiple age ranges using the STQ–FET framework. The reference to **Figure 1** that presents the components of the FET model is given only to explain the hypothesis and the results of the study. The main goal of the study was to investigate whether the differentiation between endurance, dynamical, and orientational components of temperament (as a distinct feature of the STQ and FET models) can bring new insights into the nature of depression and age differences.

### The FET Hypothesis of Temperament Profiles in MD Patients

(1) One of the main non-emotionality-related symptoms of depression is fatigue, i.e., low energetic capacities. The FET specifically distinguishes three traits on the basis of an endurance factor [Physical (ERM), Social (ERS) and Intellectual Endurance (ERI)]. If Depression manifests the symptom of fatigue through an alteration of the same regulatory systems as underlie the temperament traits related to endurance, then it is quite reasonable to expect that the presence of Depression should result in subjects reporting lower scores on these three temperament traits. Fatigue is often viewed as a general and rather non-specific factor in depression. If that is the case, then subjects in the midst of depression, particularly when fatigue is associated with a loss of motivation and drive, might experience a decline in interest toward the external world, and this may manifest as a reporting of lower scores on the orientation traits, such as Sensation Seeking, Empathy, and Sensitivity to Probability.
(2) The symptom of worthlessness in MD patients is expected to emerge as low scores on the Self-Confidence scale of the FET.
(3) Consistent with the results of many previous studies (referred to in section “Temperament and Mental Illness Lie along a Continuum”), this hypothesis predicts that reported scores on the Neuroticism scale should increase.
(4) Impulsivity is rarely discussed as a symptom of depression, however, a few studies reported high impulsivity in depressed patients (Elovainio et al., 2004; Kotov et al., 2010; Trofimova and Sulis, 2010; Trofimova and Christiansen, 2016). In line with these previous findings, a hypothesis of this study is that subjects with depression should report higher Impulsivity in comparison to healthy subjects.

### Age and Sex Specificity in the Coupling between Temperament Traits and Depression

It is well known that aging results in a progressive decline in physiological function. This leads to the rather widely held view that aging should be associated with increasing dissatisfaction with life and an elevated risk of depression. Epidemiological studies reveal a complex distribution of incidence of depression among the elderly, with large differences between independent and institutionalized elderly. In a meta-analysis, Jorm (2000) found no consistent pattern in general, although overall there appeared to be some protective effects with aging. Co-morbidities such as concurrent physical illness, single status, institutionalization, all increase the incidence (Blazer and Steffens, 2009). There have also been generational differences related to shifts in culture and in major life events which serve to distinguish individuals of different ages. The symptomatology of depression does not seem to vary much in relationship to the age of onset, although late onset depression may be more associated with cognitive dysfunction (Alexopoulos et al., 1993), medical illnesses, functional impairment (Blazer and Steffens, 2009), and psychomotor changes (such as occur in melancholia; Parker et al., 2001). In normative samples, the most consistent age-related differences in temperament traits are a decline in Motor Tempo, Social Tempo, and Sensation Seeking. It is not clear whether the presence of MD might interact with any of these factors resulting in a differential effect on reported temperament traits. Previous studies using the FET model (Trofimova and Sulis, 2010; Trofimova and Christiansen, 2016) reported a decrease in reported scores of Tempo measures and Plasticity together with higher Impulsivity scores.

From a clinical perspective, early onset and late onset depression are more alike than they are dissimilar. It is quite possible that there may be no significant interactions between age and depression. On the other hand, the association of late onset depression with greater cognitive and psychomotor dysfunction makes it reasonable to believe that depression might result in a greater impact on the temperament traits related to physical and intellectual activity (especially those describing dynamical aspects) in the old age group compared to the young and middle age groups. Predictions are hampered by the paucity of information regarding even the normative changes in temperament that manifest across the life cycle.

Many of the most common neurotransmitters involved in behavioral regulation (e.g., dopamine, serotonin, glutamate, and acetylcholine systems) change with aging (Blazer and Steffens, 2009). It is interesting to ask whether these aging changes will affect temperament. Recently a small number of longitudinal studies have started to appear in the literature (Roberts and DelVecchio, 2000; Terraccion et al., 2006; Noftle and Fleesen, 2010; Al-Halabi et al., 2011; Josefssen et al., 2013; Kandler et al., 2013). These studies have used different temperament models such as the Temperament and Character Inventory and NEO–FFI. They have found that a small number of traits such as Harm Avoidance, Novelty Seeking, Persistence do appear to change with increasing age. These models do not incorporate activity-specific traits (unlike the FET model) which are more likely to show effects as the neurobiological systems underlying vary with age. Moreover, there are virtually no studies of the impact of depression on temperament across different age groups.

Cross-sectional analysis (i.e., studies of several age groups) is the most commonly used method for studying age differences, having lower costs, and reduced time requirements in comparison to longitudinal studies. Moreover, cross-sectional studies often use contrasting age groups, such as elderly and 1825 year old participants. In this study a cross-sectional comparison was carried out using four adult age groups which is rarely seen in studies of age effects.

Our *age-related hypothesis* suggested that, if we use FET matrix of functional aspects of behavior, then age differences would be most significant in temperament traits related to the dynamics of behavior (i.e., tempo, plasticity, and impulsivity), regardless of mental illness.

It is well documented that the rates of depression among women are higher than in men. It remains unclear whether this is due to intrinsic factors such as differences in levels of, and responses to, sex hormones, or due to socio-cultural factors resulting in a bias in seeking assistance. There is neurobiological evidence that activation of κ-opioid receptor protein systems affects the level of estrogen and that this in turn lowers 5-HT, causing a stress-induced dysphoria (Filliol et al., 2000; Charmandari et al., 2005; Land et al., 2008). Low 5-HT, in turn, has been linked to dispositional emotionality disorders such as MD. This suggests that the presence of depression might exert a stronger emotional force on women than on men. If so, this might reveal itself in higher reported scores on Neuroticism and lower reported scores on Self-Confidence in depressed women in comparison to depressed men.

It is well documented that women report increased sociability compared to men. This suggests that an any interaction between gender and depression might be more noticeable in the social traits, since floor effects may minimize any impact on men. Our hypothesis in regards to *sex differences* in depression is based on well documented higher rates of depression and also sociability among women than in men which might result in lower scores on the scales measuring social-verbal and self-confidence aspects of behavior.

## Materials and Methods

### Sample

The intake records of 467 Canadians aged 17–84, patients in treatment and associates of a private psychiatric and psychological practice, Psychological Services 4018, were examined for this study. The practice serves Hamilton, Niagara Falls, Haldimand–Norfolk, and Toronto areas, and the sample represents these four distant locations. The practice has its own Late Life Memory Clinic operating under the Haldimand War Memorial Hospital (Dunnville, Ontario) that provides testing and screening for dementia in clients and patients over 60. The sample was divided into four age groups: 17–25, 26–45, 46–65, and 66–84 years old (**Table 1** for details). Subjects were included into the MD subgroup if they met the criteria for a diagnosis of MD. This diagnosis was based on the structured DSM-IV clinical interview, file review and the results of testing using the Beck Depression Inventory (scores of 36 or higher), Hamilton Depression Inventory (scores of 20 or higher on the Total scale), and Symptom CheckList-90 (scores of 40 or higher on the Depression scale). Two hundred twenty-six patients (M/F = 102/124) were included into the MD subgroup. Data from the patient groups was compared to data from a control group of 241 clients or volunteers (M/F = 82/118). This control group included volunteers and people who were referred to Psychological Services but who did not meet the criteria for MD. Subjects were excluded from both control and MD groups if they had a history of bipolar illness, generalized anxiety disorder, dementia or were exhibiting symptoms of hypomania or mania.

**Table 1:** Information about the sample and the groups of the study.

Groups	Sample				MD group		non-MD group	
			
	*Age*	*M*_age_ *(SD)*	*total*	*M/F*	*N*	*M/F*	*N*	*M/F*
Sample	17–84		467	205/262	226	102/124	241	82/118
Age1	17–25	19.86 (2.57)	136	55/81	44	13/31	92	42/50
Age2	26–45	35.60 (5.77)	130	68/62	74	44/30	56	24/32
Age3	46–65	54.57 (5.35)	130	48/82	75	27/48	55	21/34
Age4	66–84	76.80 (5.87)	71	34/37	33	18/15	38	16/22


The forms and the method of the study were approved by the Hamilton Integrated Research Ethics Board (McMaster University), Hamilton, ON, Canada.

The sample of MD had sex and age ratios reflective of those in clinical sub-populations of patients diagnosed with this disorder in Canada. Thus, females use medical (including psychological) services more often than males, so our sample had more females than males, and the control samples matched the sex ratios of the experimental samples, for purposes of statistical processing. Similar considerations were applied to marital status, education, and socio-economic status of the participants. The factor of Age was analyzed using contrast-group statistics and therefore balance between age groups was more important than ensuring a correspondence of the sample age distribution with the age distribution within a general population. Moreover, the youngest age group has significantly lower occurrence of MD in the general population than other adult age groups, and this was reflected in the smaller size of this group in our study (**Table 1**).

### Procedure and Measures

All participants of this study signed a consent allowing the use of their intake forms for research purposes. During either intake testing (for patients and clients) or research (for healthy participants) each person completed the Compact STQ-77 (Rusalov and Trofimova, 2007; Trofimova, 2010a,b; Trofimova and Sulis, 2011)^1^. The STQ-77 consists of 77 statements, assigned to 12 temperament scales (six items each) and a validity scale (five items, addressing social desirability bias), which are listed below. Subjects responded according to a 4-point Likert scale format: (1) “strongly disagree,” (2) “disagree,” (3) “agree,” (4) “strongly agree.”

The temperament scales are organized in groups as following (**Figure 1**):

1-3: Endurance group – the scales of Physical, Social, and Intellectual Endurance: the ability of an individual to sustain prolonged physical (ERM, alpha Cronbach for this data = 0.82), social (ERS, α = 0.77), or mental (ERI, α = 0.72) activity.
4-5: Dynamic group – the scales of Physical Tempo (preferred speed of physical activity; TMM, α = 0.81), Social Tempo (speed of speech and reading and of other verbal activities; TMS, α = 0.70), and the Plasticity scale (assessing the ability to adapt quickly to changes in situations, to change the program of action, and to shift between different tasks; PL, α = 0.75).
6-9: Sensitivity group – assessing how much behavior of an individual is oriented primarily to risky or physical pleasures (Sensation Seeking scale, SS, α = 0.76), to other people’s emotional state (Empathy scale, EMP, α = 0.67), and to probabilistic processing of causes and consequences of events (Sensitivity to Probabilities scale, PRO, α = 0.70).
10-12: Emotionality group – Self-confidence scale (SLF, α = 0.72), the tendency to be optimistic and confident (sometimes overly optimistic) in one’s own performance, to ignore other people’s warnings and criticism; Impulsivity scale (IMP, α = 0.71) as emotional reactivity, a poor ability to control immediate impulses for actions; Neuroticism scale (NEU, α = 0.70), low tolerance of uncertainty and novelty, negativity bias in expectations of outcomes in own activity.
13 Validity scale – social desirability tendency in answers. Results within the range of 15–20 on the validity scale should be considered invalid as the respondents are likely to demonstrate positive impression bias in their responses.

### Statistical Processing

Statistical processing included calculation of the descriptive scale statistics for the whole sample and separately by age and sex groups. The means of the four age groups and two sex groups on the STQ scales were also submitted to one-way and factorial analysis of variance to examine the impact of age and sex factors in depression and temperament (with AgeGroup, Sex, and Diagnosis (Normal vs. Depressed) as a parameters). *Post hoc* comparisons were performed using both the Tukey and Fisher LCD tests with an alpha level of 0.05. The partial Eta-squared values (η^2^) were also calculated as an additional metric of effect size for all significant ANOVA contrasts.

## Results

**FIGURE 2:**
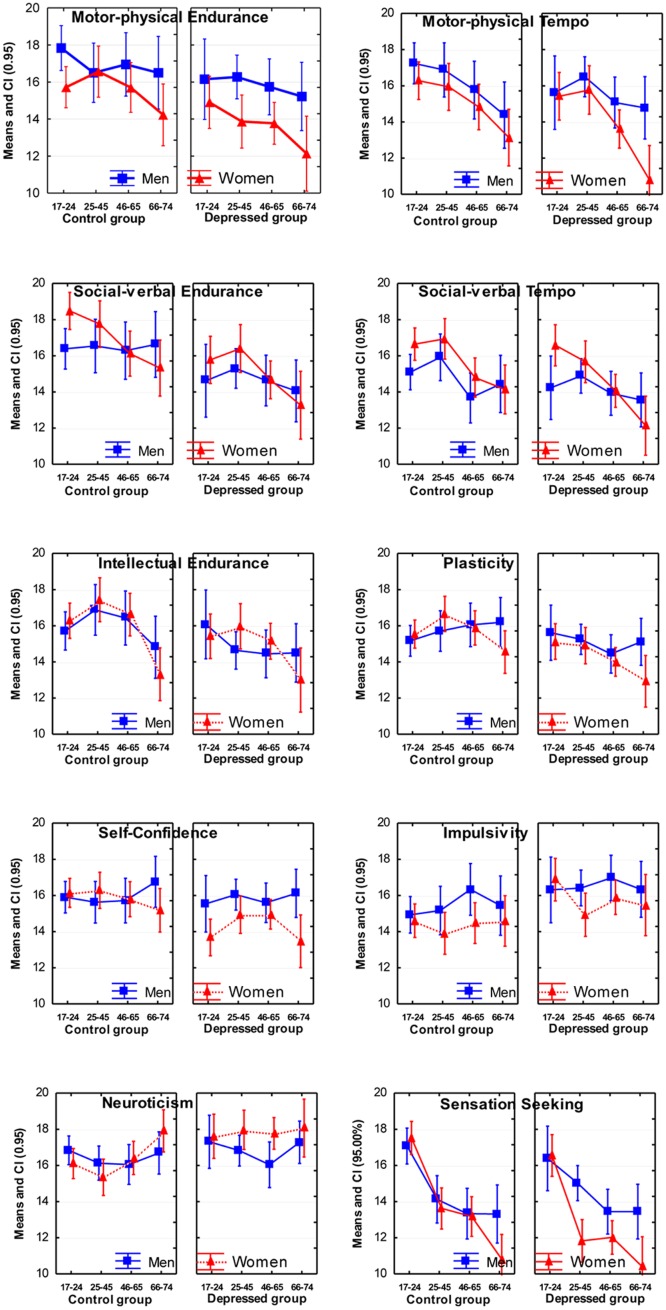
**Means and Confidence Intervals (CI; 95%) of the scores on temperament scales of the Structure of Temperament Questionnaire (STQ)-77**.

Depression showed significant effects on nine out of 12 traits (**Table 2**; **Figure 2**): a decrease in Motor-physical Endurance, Motor-physical Tempo, Social Endurance, Social Tempo, Intellectual Endurance, Plasticity, and Self-Confidence. Impulsivity and Neuroticism increased in depression.

**Table 2:** Means and Standard Error (*M*_SE_) on the Structure of Temperament Questionnaire (STQ)-77 scales for groups contrasted by depression (all ages combined), and the ANOVA effects.

STQ-77	Controls *N* = 241	Depres-d *N* = 226	Effect of Depression		
		
scales	*M*_SE_	*M*_SE_	*F*(1,465)	*p*	η^2^
Motor-physical Endurance	16.25_0.27_	14.76_0.29_	13.84	0.000	0.030
Motor-physical Tempo	15.55_0.26_	14.69_0.27_	5.26	0.022	0.012
Sensation Seeking	14.12_0.22_	13.64_0.24_	2.18	0.140	0.005
Social-verbal Endurance	16.69_0.25_	14.84_0.27_	24.83	0.000	0.052
Social-verbal Tempo	15.20_0.22_	14.38_0.24_	6.47	0.011	0.014
Empathy	17.11_0.17_	17.03_0.17_	0.04	0.841	0.000
Intellectual Endurance	16.09_0.21_	14.97_0.25_	8.75	0.003	0.019
Plasticity	15.68_0.17_	14.68_0.20_	13.74	0.000	0.030
Sensitivity to Probabilities	16.53_0.21_	16.09_0.23_	2.14	0.144	0.005
Self-confidence	15.93_0.17_	15.09_0.21_	9.36	0.002	0.020
Impulsivity	14.81_0.20_	16.16_0.24_	13.20	0.000	0.028
Neuroticism	16.37_0.17_	17.34_0.20_	10.97	0.001	0.024


*Zeros at *p*- and η^2^ values are omitted.*

In terms of age effects, the most significant effects were lower scores reported by normal subjects in the older age groups on the scales of Motor Tempo, Social Tempo, Sensation Seeking and Intellectual Endurance, and weaker, but still significant effects on the scales of Social-verbal Endurance and Sensitivity to Probabilities. There were no significant interactions effects between Age and Depression (**Table 3**).

**Table 3:** Means and Standard Error (*M*_SE_) on the STQ-77 scales for groups contrasted by Age, and the ANOVA effects.

STQ-77	Age1 *N* = 136	Age2 *N* = 130	Age3 *N* = 130	Age4 *N* = 71	Effect of Age		
		
scales	*M*_SE_	*M*_SE_	*M*_SE_	*M*_SE_	*F*(3,463)	*p*	η^2^
ERM	16.15_0.39_	15.80_0.36_	15.54_0.37_	14.52_0.48_	2.48	0.061	0.016
TMM	16.14_0.36_	16.27_0.33_	14.82_0.34_	13.27_0.45_	12.11	0.000	0.075
SS	16.88_0.32_	13.65_0.29_	12.99_0.30_	11.99_0.39_	40.09	0.000	0.211
ERS	16.31_0.36_	16.50_0.33_	15.42_0.34_	14.82_0.44_	4.16	0.006	0.027
TMS	15.64_0.31_	15.85_0.29_	14.11_0.30_	13.57_0.39_	11.80	0.000	0.073
EMP	17.14_0.26_	17.28_0.21_	17.02_0.23_	16.68_0.29_	1.06	0.364	0.007
ERI	15.89_0.28_	16.05_0.33_	15.59_0.32_	13.89_0.42_	7.03	0.000	0.045
PL	15.34_0.22_	15.60_0.25_	14.92_0.29_	14.72_0.32_	1.85	0.138	0.012
PRO	16.40_0.28_	16.32_0.29_	16.72_0.29_	15.44_0.40_	3.01	0.030	0.020
SLF	15.45_0.23_	15.77_0.28_	15.43_0.26_	15.41_0.35_	0.43	0.729	0.003
IMP	15.38_0.30_	15.22_0.29_	15.82_0.32_	15.41_0.36_	1.28	0.280	0.008
NEU	16.79_0.25_	16.59_0.25_	16.78_0.26_	17.51_0.30_	2.10	0.099	0.014


*Age1: 17–25; Age2: 26–45; Age3: 46–65, Age4: 66–84. Zeros at *p*- and η^*2*^ values are omitted.*

Women reported significantly lower scores in Motor Endurance, Motor Tempo, Sensation Seeking, Self-Confidence, Impulsivity, and Sensitivity to Probabilities compared to men, and higher scores on Social Tempo (**Table 4**). There was a significant Sex × Depression interaction effect on the scale of Self-Confidence (*F* = 5.99; *p* = 0.015; η^2^ = 0.013). *Post hoc* analysis showed that depressed women reported significantly lower scores in Self-Confidence compared to depressed men.

**Table 4:** Means and Standard Deviations (*M*_SD_) on the STQ-77 scales for groups contrasted by Sex (all Men combined vs. all Women combined), and the ANOVA effects.

STQ-77 Scales	Men *N* = 205	Women *N* = 262	Effect of Sex		
			
			*F*(1,387)	*p*	η^2^
Motor Endurance	16.40_0.30_	14.61_0.26_	19.82	0.000	0.042
Motor Tempo	15.78_0.28_	14.47_0.25_	12.17	0.001	0.026
Sensation Seeking	14.52_0.25_	13.24_0.22_	15.06	0.000	0.032
Social Endurance	15.55_0.28_	15.97_0.24_	1.27	0.261	0.003
Social Tempo	14.46_0.24_	15.12_0.21_	4.09	0.044	0.009
Empathy	16.93_0.16_	17.19_0.18_	0.61	0.437	0.001
Intellectual Endurance	15.37_0.24_	15.68_0.23_	0.01	0.914	0.000
Plasticity	15.35_0.18_	15.08_0.19_	3.07	0.081	0.007
Sensitivity to Probabilities	16.91_0.22_	15.85_0.21_	16.43	0.000	0.035
Self-confidence	15.90_0.19_	15.23_0.19_	8.79	0.003	0.019
Impulsivity	15.94_0.21_	15.09_0.23_	7.09	0.008	0.015
Neuroticism	16.62_0.18_	17.01_0.19_	3.28	0.071	0.007


*Zeros at *p*- and η^*2*^ values are omitted.*

There were also two Sex × Age interaction effects: on the scale of Sensation Seeking (*F* = 3.74; *p* = 0.011; η^2^ = 0.024) and Social-verbal Tempo (*F* = 2.69; *p* = 0.046; η^2^ = 0.018). On these scales older women reported lower scores much more significantly than did older men.

## Discussion

### General Effects of Coupling of Depression with Temperament Traits

If temperament and mental illness, such as MD, share common causes, then mental (and physical) illness could shift biological systems of behavioral regulation. This would facilitate the development of such traits as impulsivity, neuroticism, low sociability, low energetic level, etc. Alternatively, some temperament profiles (such as low endurance, high neuroticism) could create a disposition for a mental disorder (Clark, 2005). In this sense it would be more accurate to talk about a *coupling* of temperament traits with mental illness rather than about causal relationships.

The results of this study showed a good correspondence between the DSM/ICD symptoms of MD and expected effects in the STQ profiles in MD patients:

- A pervasive decline in the energetic group mirroring the depressive symptom of fatigue, impacting on Physical and Social Endurance traits;
- A decline in Intellectual Endurance in line with the DSM/ICD symptom of poor concentration in MD;
- A decline in Physical Tempo and Social-verbal Tempo traits, in line with the DSM/ICD symptom of psychomotor retardation;
- Lower scores of MD patients on the scale of Self-Confidence, in comparison to non-MD participants, in line with the DSM/ICD symptoms of worthlessness and low mood in MD.

These declines held across all age groups and both sexes, supporting the universality of these five symptoms of depression. Moreover, the changes noted in the presence of MD contrasted dramatically with those previously reported in Generalized Anxiety Disorder, where only four traits showed effects (decreased Social Endurance, Social Tempo, and Sensitivity to Probabilities, increased Neuroticism; Trofimova and Sulis, 2016a).

The neurobiology of fatigue is complex, involving dopamine, orexin, and partly mediated via disruption of circadian systems, which also involve serotonergic, and noradrenergic systems (Krishnan and Nestler, 2010; Harrington, 2012). The serotonin system has long been implicated in endurance aspects of both mental and physical performance although the relationship is far from simple (Struder and Weicker, 2001). Opioid receptor systems regulate the release of monoamines, and their up- or down-regulation can trigger a dysregulation of monoamines linked to depression (Johnston and North, 1992; Comer et al., 1993; Degli-Uberti et al., 1995; Jutkiewicz et al., 2005). The results are consistent with the FET model which suggests that alterations in opioid receptor activity induce dysfunctions in monoamine and orexin systems, and as a result one should observe a decrease in the endurance of behavior in all three FET domains: physical, social (sociability), and intellectual (concentration and attention). Emotionality-related symptoms of MD are a feeling of worthlessness and dysphoria, linked to upregulation of MOPr (Bloom, 1983), and to subjective affectivity (Degli-Uberti et al., 1995; Bodnar, 2011; Lutz and Keiffer, 2013).

The selectivity of depression is most notable in the lack of effect on the orientation group of traits (Sensation Seeking, Empathy, and Sensitivity to Probabilities). The negative cognitive bias associated with depression would be expected to emerge as significant differences on these traits between MD and non-MD patients, yet this was not observed. This is an important finding, pointing to possible difficulties in using CBT that focuses on cognitive constructs in depressed patients’ perception (i.e., orientational rather than executive aspects of behavior). It suggests that behavioral orientation capacities might not be affected by MD but are deactivated by weak executive systems (i.e., the negative impact of lower endurance, slower tempo, behavioral rigidity, and low self-esteem).

An increase in Neuroticism scores was expected given the large number of previous studies showing such an effect (Costa et al., 2005; Kotov et al., 2010; Klein et al., 2011). This was indeed the case. In spite of similar associations between Neuroticism and MD as reported in several studies, neuroticism is still not in the current list of DSM/ICD symptoms of MD. Our finding here suggests that it should be considered for inclusion in future versions of the DSM.

An increase in Impulsivity in the presence of depression is opposite to what would be expected in the presence of fatigue or psychomotor retardation. Clinicians would expect higher impulsivity in bipolar patients, however, depressed patients in this sample were screened for bipolarity and did not have a history or symptoms of hypomania or mania. This result is consistent, however, with previous studies (Elovainio et al., 2004; Kotov et al., 2010; Trofimova and Sulis, 2010; Trofimova and Christiansen, 2016). Moreover, these findings are in line with the FET model (**Figure 1**) that suggests that an interaction of DOPr with dopamine and MOPr systems appears to play an important role in the speed of generation of an action, whether adequate (such as Tempo of learned actions, or Plasticity in generation of new actions) or premature (i.e., impulsivity; Comer et al., 1993; Broom et al., 2002; Jutkiewicz et al., 2005; Olmstead et al., 2009; Pradhan et al., 2011). In depression, the integration of behavior can be compromised due to the impact of a dysregulation of MOPr and DOPr systems on DA release. Behavior could become not only sluggish, but also less plastic. At the same time it could become over-reactive toward occasional stimuli that would not normally trigger a behavioral reaction, leading to an elevation of impulsivity. An increase in reported Impulsivity scores and decreases in Plasticity and Tempo were indeed observed in our study.

An elevation of Impulsivity and a decrease in Plasticity may represent new symptoms of depression which were not previously differentiated from psychomotor retardation but which warrant consideration for subsequent versions of the DSM/ICD.

The value of the FET model is two fold. It differentiates between traits on the basis of activities, which permits a mapping between certain DSM symptoms and temperament traits. It is also sensitive to the presence of mental illness, resulting in differential effects on temperament profiles in MD and generalized anxiety [Trofimova and Sulis, 2016a,b; and possibly other illnesses (Trofimova and Christiansen, 2016)]. This is a significant advantage over other temperament models which have shown a limited ability to distinguish between these two illnesses. This supports our contention that the use of a framework for describing behavioral regulation such as that provided by the FET model provides a unifying approach to the classification and study of both temperament and mental illness. This is consistent with the view that they form opposite extremes along a continuum of behavioral regulation.

### Sex Differences in Temperament

In the presence of depression, men and women appeared to report remarkably similar effects on temperament profiles. This is consistent with our view that depression serves as a general modulator of traits. Depressed women, however, reported significantly lower scores for Self-Confidence than their healthy counterparts. It is possible that the higher rates of depression reported by women in comparison to men are a reflection of depression-related changes in their self-confidence rather than in energetic level.

In this sample, women generally reported significantly higher scores for Social Endurance (sociability) and Social Tempo than men, in line with the literature (Trofimova, 2015). There are suggestions that self-confidence in women may be related to perceived social supports (Nolen-Hoeksema, 2001). The endogenous MOPr system that is, according to the FET hypothesis, one of the main players in MD, was found to be important in social perceived support (Way et al., 2009) and affiliative behavior (Keverne and Curley, 2004; Depue and Morrone-Strupinsky, 2005; Barr et al., 2008). It is also involved in the regulation of oxytocin that is even more commonly implicated in sociability and perceived social support (Bielsky and Young, 2004; Depue and Morrone-Strupinsky, 2005; Taylor et al., 2006; Donaldson and Young, 2008; Barraza and Zak, 2009). The loss of oxytocin function may result in a perception of diminished social support, which in turn may lead to a decrease in Self-Confidence. It is important to note that there was no significant difference between healthy men and women with regards to reported levels of Self-Confidence and Neuroticism, contradicting the popular view that women are more neurotic than men.

Regardless of the presence of depression, men reported significantly higher scores for Physical Endurance, Physical Tempo, Sensation Seeking, and Sensitivity to Probabilities compared to women, consistent with well known physical differences between the sexes and with higher rates of risk- and sensation seeking, criminal behavior, and openness to experience in males than females (Zuckerman, 1994; Costa et al., 2001; Trofimova, 2015).

These sex differences are subtle and can be easily identified when temperament traits are differentiated into physical and social aspects of behavior, as well as between endurance and tempo of actions. These results again demonstrate the usefulness of such a differentiation in the FET model.

### Age Differences in Coupling of Depression and Temperament Traits

It is reasonable to think that aging should influence the way in which depression impacts on the temperament system. This, however, proved not to be the case. As an important negative result, there were no significant interaction effects between the presence of depression and age. This result suggests that the deleterious effect of depression on temperament profiles are universal across all age groups.

Some general effects of age on temperament were observed, with or without depression. The findings of lower scores on Tempo-related scales and on Sensation Seeking in older groups, in comparison to younger groups, are in line with the current literature reporting a slowing of processes with age (Birren and Schaie, 2001) and a decrease in Sensation Seeking (Zuckerman, 1994).

Intriguing are changes between the young and middle age groups when there is an increase in the means for Intellectual Endurance and Plasticity (PL) and a decrease for Impulsivity (IMP) and Neuroticism (NEU). The last two effects were consistent with previous studies (Roberts et al., 2006). This is conjectured as being most likely due to maturation of the frontal lobes, which is known to be complete around age 24. ERI and PL measure dynamical characteristics of the intellectual (i.e., frontal) systems, and so the immaturity of these frontal systems could be expected to result in a lesser ability to sustain intellectual activity (ERI) and to exercise flexibility under changing conditions (PL). Maturation of the frontal lobes results in the achievement of a maximal capacity for intellectual activity after age 25, and this is reflected in the larger scores obtained in the 25–45 age group. The middle age group reaps the benefits of this frontal maturity by having greater intellectual endurance and plasticity, and by being less impulsive and neurotic. These gains subside with increasing age, so that the old group comes to look more like the young group, with less ERI and PL, and more IMP and NEU.

Most interesting is that if these groups are split by sex, then it appears that it is women who benefit most from the early maturation of the frontal lobes. They account for the bulk of the increase in ERI and PL and the decrease in IMP and NEU. Indeed men showed little difference between young age and middle age in these traits. This effect in women is in the 25–45 age group and it is remarkable that this coincides precisely with the time during which women must take on multiple roles as wife, parent and often worker, requiring extensive multitasking and endurance. This suggests that hormonal factors, or at least social factors, might also play a role in stimulating this additional frontal lobe development.

These subtle interaction effects between age and gender in healthy subjects again point to the usefulness of differentiating between traits based upon their functionality (endurance, dynamical, or orientation).

## Conclusion

The FET framework attempts to provide a correspondence between the clinical taxonomy of mental disorders and a taxonomy of individual differences in healthy people (i.e., temperament). The commonality of etiology between mental disorders and temperament (presumably sharing the same underlying neurotransmitter systems) encouraged several psychiatrists to search for models and theories uniting these taxonomies, and this paper follows this research tradition.

This study investigated the discriminative ability of the FET framework which differentiates between physical, social, and mental aspects of behavior as well as between orientation, dynamical, and endurance-related aspects. The results of this study showed the benefits of such a differentiation as applied to the structure of symptoms of MD, as well as in the analysis of age and sex differences:

(1) The connection between temperament traits and DSM symptomatology was through the mapping of five DSM/ICD symptoms of MD (feeling of worthlessness, low mood, fatigue, poor concentration, and psychomotor retardation) to the FET matrix. In line with the hypothesis, scores on the scales of Self-Confidence, Physical Endurance, Social Endurance, Intellectual Endurance, Physical Tempo, and Social Tempo were significantly lower in MD patients.
(2) The FET hypothesis also predicted a coupling between MD and three other temperament traits (lower Plasticity, higher Impulsivity, and Neuroticism) that do not have analogs in the DSM/ICD symptoms of MD. This hypothesis was also supported. Impulsivity and low Plasticity are not equivalent to restlessness or psychomotor agitation and should receive separate emphasis. Elevations on the traits of Impulsivity and Neuroticism together with a decrease in the trait of Plasticity may be a risk factor for MD. Further clinical studies are needed to determine whether this has clinical utility.
(3) Consistent with the literature, sex differences (regardless of the presence of MD) were found in temperament traits differentiating between physical and social aspects of behavior. Men reported significantly higher scores for Physical Endurance, Physical Tempo, Sensation Seeking, and Sensitivity to Probabilities compared to women. Women reported higher scores on Social Tempo. These results illustrate the benefits of differentiating between physical and social aspects of activities.
(4) The decline in Self-confidence was more significant in women than in men in the presence of MD, but no sex differences in the effect of MD on nine functional aspects of behavior were found. This suggests that sex differences in depression lie more in emotional regulation rather than in performance capacities.
(5) The coupling between depression and temperament was analyzed across four different age groups. As a significant negative result, no interaction of age with MD was found for temperament profiles. This result suggests that the deleterious effects of depression on temperament profiles reflecting both emotional regulation and performance capacities appear to be universal across all age groups.
(6) Consistent with the literature, age differences (regardless of the presence of MD) were found in temperament traits differentiating between dynamical (speed of integration of an action) and endurance aspects of behavior. Non-depressed participants aged 66–84 had significantly lower scores on Physical Endurance, Social Tempo, Sensation Seeking, and Intellectual Endurance, in comparison to younger age groups. Participants aged 46–65 had significantly higher means on the scales of Intellectual Endurance and Plasticity and lower Impulsivity and Neuroticism, in comparison to younger and older participants.

Overall, the results of this study suggest the benefits of differentiating between emotionality and non-emotionality-related traits based on the functionality of neurotransmitter systems. They point to the utility of using a functional approach to both the taxonomy of temperament and the classification of mental disorders. They also demonstrate the benefits of differentiating between the 12 aspects of behavior as mapped in the FET framework.

This lends support to the idea of using a model of behavioral regulation (such as the FET model) as a basis for the classification of mental disorders. The effects of depression on FET temperament traits also suggest considering the addition of new diagnostic symptoms of depression-related to plasticity, impulsivity, and neuroticism.

The limitations of this study relate to the self-report nature of the STQ-77 used in this study and the cross-sectional character of the analysis of age differences. However, the self-report format is standard in temperament research and in psychiatric diagnostic practice, and cross-sectional studies are the most common methods in the investigation of age differences. The STQ Validity scale helped to minimize the effect of positive impression bias on data of this study.

## Author Contributions

IT designed the study, wrote the protocol, had a partial literature search, performed statistical analysis, and wrote a first draft of the manuscript. WS contributed to the literature searches, significantly edited “Introduction” and “Discussion” sections of the manuscript, and managed formatting of the references. All authors have approved the final manuscript.

## Conflict of Interest Statement

The authors declare that the research was conducted in the absence of any commercial or financial relationships that could be construed as a potential conflict of interest.

## Footnotes

1 The complete validation history can be found in Rusalov and Trofimova (2007); Trofimova (2010a,b).
